# Correlated Observations, the Law of Small Numbers and Bank Runs

**DOI:** 10.1371/journal.pone.0147268

**Published:** 2016-04-01

**Authors:** Gergely Horváth, Hubert János Kiss

**Affiliations:** 1 Department of Economic Theory, Friedrich-Alexander-Universität Erlangen-Nürnberg, Nuremberg, Bavaria, Germany; 2 Department of Economics, Eötvös Loránd University and Momentum(LD-004/2010) Game Theory Research Group at MTA KRTK, Budapest, Hungary; Universidad de Alicante, ITALY

## Abstract

Empirical descriptions and studies suggest that generally depositors observe a sample of previous decisions before deciding if to keep their funds deposited or to withdraw them. These observed decisions may exhibit different degrees of correlation across depositors. In our model depositors decide sequentially and are assumed to follow the law of small numbers in the sense that they believe that a bank run is underway if the number of observed withdrawals in their sample is large. Theoretically, with highly correlated samples and infinite depositors runs occur with certainty, while with random samples it needs not be the case, as for many parameter settings the likelihood of bank runs is zero. We investigate the intermediate cases and find that i) decreasing the correlation and ii) increasing the sample size reduces the likelihood of bank runs, *ceteris paribus*. Interestingly, the multiplicity of equilibria, a feature of the canonical Diamond-Dybvig model that we use also, disappears almost completely in our setup. Our results have relevant policy implications.

## 1 Introduction

Although bank runs were very rare phenomena in developed countries in the decades before 2007, the run on Northern Rock, an English bank, heralded that“… [*T*]he age of the bank run has returned.” (Tyler Cowen, The New York Times March 24, 2012) Episodes of banks and other financial institutions suffering sudden and massive withdrawals of deposits and other funding sources were frequent during the recent financial crisis (e.g. the investment bank Bear Stearns in the US, the DSB Bank in the Netherlands or Bankia in Spain). Deteriorating fundamentals are a prime cause of bank runs, but there is often also a substantial self-fulfilling component to the behavior of depositors. Depositors hurry to withdraw fearing that other depositors’ withdrawal will cause the bank to fail. This idea is illustrated vividly by the words of Anne Burke, a client of Northern Rock, who said the following while queuing up to withdraw her funds: “It’s not that I disbelieve Northern Rock, but everyone is worried and I don’t want to be the last one in the queue. If everyone else does it, it becomes the right thing to do.”(See http://www.bloomberg.com/apps/news?pid=newsarchive&sid=aeypCkzcRlU4) The above quote shows that depositors react to other depositors’ observed or known decisions. Empirical studies ([1]; [2]; and [3]) and experimental findings ([4]; [5]) support this idea as well.

It is natural to ask: what do depositors observe or know about previous decisions? In some cases observability of other depositors’ actions is almost non-existent, as it was the case during the silent run on Washington Mutual in 2008, when depositors withdrew their funds electronically. When nothing is observed, [6] show experimentally that bank runs are more likely the more stringent are the conditions for the coordination of depositors. Other empirical observations suggest two things. First, although it is easier to observe somebody queuing up to withdraw, depositors may also know that others have decided to keep their money deposited. [1] and [3] point at the importance of observing decisions of both sorts (withdrawal or keeping the money in the bank). [2] argue that during a bank run incident in 2001 in Turkey small and medium-sized depositors seemed to observe only withdrawals of their peers but the behavior of large depositors appeared to be driven by observing both choices. [7] in chapter 9 cites ample evidence about how interpersonal and word-of-mouth communication affects financial decision-making. Hence, even if you cannot observe somebody deciding to keep her funds in the bank as generally it does not involve any special action, through communication one may get to know that somebody made that decision. Second, the previous empirical studies and other descriptions suggest that not all previous decisions can be observed, depositors observe only a sample of earlier choices.

A noteworthy aspect of the above empirical studies is that none of the banks affected by the runs were fundamentally bad banks. Thus, the massive withdrawals cannot be explained by the decisions of informed depositors withdrawing from a financial intermediary due to fundamental reasons, but a coordination failure among the depositors seems to be behind these runs. It is of first-order importance to understand what may cause these coordination failures since it is clearly not optimal that healthy banks suffer bank runs by depositors and the financial intermediation is disrupted. Although fundamentally weaker banks are more likely to be affected by bank runs, there is clear evidence (e.g. [8]; [9]) that even fundamentally healthy financial intermediaries experience excessive withdrawal episodes.

In this study we attempt to further our understanding by assuming a healthy bank that has depositors deciding in a sequential manner and who act upon observing a sample of previous decisions. The main question that we study is how sampling affects the emergence of bank runs. In close-knit communities (for instance, among customers of a rural bank in a village) the information observed by subsequent depositors generally overlaps, depositors who decide sequentially observe to a large degree the same previous choices. On the contrary, for clients of a big bank information is less correlated, depositors deciding consecutively are less likely to observe the same previous decisions. Do these differences lead to a different probability of bank run?

To answer this question we develop a model in which depositors observe a sample of the previous actions and decide sequentially. For simplicity, we consider the case when depositors observe samples of the same size. We change the degree of correlation across subsequent samples. A key issue is how depositors extract information from the observed sample. It is not obvious what conclusion a depositor can draw when observing a set of previous decisions that need not be representative and knowing that her decision will be possibly observed by others and hence influence if a bank run starts or not. We use a well-known regularity of decision-making documented in behavioral economics: the law of small numbers (see [10] and [11, 12] provide related examples in finance). It is a faulty generalization, the observed sample is taken to be representative of the population of interest. Hence, if a depositor observes many withdrawals (that is, above a threshold) in her sample, then she believes that the number of withdrawals in general will be large so withdrawing early may seem the optimal decision since otherwise the depositor may receive nothing from the bank. On the contrary, when only a few withdrawals are observed, it may suggest that there is no bank run underway, so there is no reason to withdraw the money from the bank. The law of small numbers relies on the availability and representativeness heuristics and leads to overinference, that is the belief that even small samples reflect the properties of the population ([13] and [14] are two important studies on the availability and the representativeness heuristics.)

We study two focal cases. In the random sampling case, depositors observe any of the previous decisions with equal probability, so correlation across consecutive samples is low. With overlapping samples, depositors observe the last decisions. This sort of sampling captures several intuitive ideas. On the one hand, it can be argued that recent actions are more likely to be observed. On the other hand, depositors with the same characteristics (those living in the same neighborhood as in [1] or the depositors grouped according to their deposit size as in [2]) tend to observe the same information. Our overlapping sampling is a way to have depositors observe highly correlated information. The intuition why different sampling mechanisms lead to different results is the following. With overlapping samples subsequent depositors observe very similar samples, so if a depositor observes a sample that makes her withdraw, the next depositor will observe at least as many withdrawals as the last one. If depositors follow identical decision rules, then it leads to a bank run. With random sampling the correlation across samples is considerably lower, so subsequent decisions will be less uniform resulting in less bank runs.

As a comparative statics exercise, we also analyze the intermediate cases where we systematically change the fraction of the sample that is random and the fraction of the sample that can be correlated. We find consistently that the correlation of the samples affects the probability of bank runs, higher correlation leading to an increased probability of bank runs, *ceteris paribus*. As we decrease the correlation of samples, the probability of bank runs decreases in a non-linear way, suggesting a sharp transition from a state where bank runs are very likely to another where they do not occur. In the simulations we find also that as the sample size increases, the probability of a bank run decreases, other things being equal. In the paper we try to delineate the conditions that lead to a certain bank run and also to show which conditions result in no bank run. One of the main findings is that for a wide range of parameter values the outcome is clear, so the multiplicity of equilibria that characterizes the Diamond-Dybvig framework does not occur generally in our setup.

Our results suggest how banks and policy-makers could prevent bank runs that are unjustified fundamentally and are triggered by overly panicky depositors. By providing more information about previous decisions (that is, increasing the sample size) and by attempting to make that information the least correlated, the probability of bank runs can be diminished. Banks can decrease the correlation in information across depositors by diversifying their depositor pool, that is, instead of focusing on a determined community they should serve different groups. The threshold in the decision rule of the depositors can also be affected by credible policies and the safety network (e.g. deposit insurance).

The rest of the paper is organized as follows. In section 2 we review briefly the relevant literature. Section 3 presents the model and it contains the results for overlapping and random sampling. Section 4 has the simulation results for the intermediate cases and section 5 concludes.

## 2 Related Literature

In this section we argue that i) depositors react to the sample of previous decisions that they observe; ii) the law of small numbers describes fairly well how depositors react to what they observe, and iii) correlation across samples matters also. We finish the section discussing briefly the connection to the literature on learning and diffusion on social networks.

Most theoretical papers assume that depositors make the decision about withdrawal of funds simultaneously (e.g. [15]; [16]). A notable exception is [17] who suppose that depositors decide after each other according to a predetermined order and they observe *all* previous choices before making decision. They study two setups: when, besides actions, liquidity needs of previous depositors are also observed, and when only the previous actions are observed and liquidity needs are private information. In both cases, they obtain that bank runs do not occur in equilibrium.

In this study we suppose that depositors do not observe the decisions of all depositors preceding them, but only a subset. Do people rely on partial information provided by a sample? [1] test a contagion model which focuses on the banking panics in a New York bank in 1854 and 1857 based on the connections between Irish immigrants. They find that although there were also other factors at work, the most important one determining whether an individual panicked was his county of origin in Ireland. Almost identical persons, only differing in the county of origin, behaved differently during the panics, and the opinions of others with the same background and their choices had the most influence on the decisions of their peers. The origin had its effect through the fact that inmigrants from the same county tended to live in the same neighborhood and observed each other. [3] show that in a bank run episode that took place in India in 2001 a depositor’s probability of withdrawing was increasing in the share of other people in her neighborhood or among her introducers who had done so earlier (in India, to open an account banks may require that the person be introduced by someone who already has an account in the bank). Clearly, a depositor’s social connections or neighborhood comprise only a reduced subset of all depositors, so only a sample of previous decisions affected if a depositor chose to withdraw or not. [5] find in an experiment that observing that some (but not all) previous depositor has (not) withdrawn increases (decreases) significantly the probability of withdrawal.

As already mentioned, the law of small numbers means that agents expect small samples to exhibit large-sample statistical properties, so people tend to overweigh information that is available. The main cause behind this phenomenon is that people are too inattentive to the sample size, so they tend to draw overstretched inferences based on small samples (for examples see section 2 in [10]). Evidence of people falling prey to the law of small numbers and consequently to overinference abounds when the sample and the population outcome is generated by an exogenous device (e.g. flipping a coin, guessing the urn from which a ball was drawn), but there is less evidence when the observed sample is the outcome of other individuals’ decision. However, the findings in [1] and [3] suggest that depositor behavior can be rationalized by the law of small numbers. More precisely, individuals observing a low number of withdrawals among their peers in their sample may believe that overall the number of withdrawals will be low, so there is no need to run the bank. By the same token, observing many withdrawals in a sample may cause individuals to think that there is a bank run underway. We do not claim that this is the only or the best explanation for their behavior, but it seems a reasonable one. This suggests that a threshold rule may reflect the law of small numbers when studying depositor behavior. In a bank-run setup, [4] explore experimentally the coordination problem among depositors and find that simple cutoff rules explain fairly well the behavior of the participants.

The arguments in [1] suggest that correlation of observed behavior is important as people in the same neighborhood had highly overlapping information and they acted also in a very similar way. [3] also evoke the relevance of correlated samples as depositors in the same neighborhood may observe to a large degree the same previous decisions. Moreover, several depositors may have the same introducer that also may make information correlated. Such overlapping of information may be due to clustering, one of the key empirical regularities found in social networks. Clustering refers to the tendency of linked nodes to have common neighbor(s) (for more details see for instance [18] or [19]). That is, a depositor observes what her neighbors do and at the same time those neighbors are likely to observe each other. [20] studies the effect of radio penetration on bank runs and banking distress during the Great Depression. He finds that a 10-percentage point increase in radio penetration in a county resulted in a 4.4 percentage point fall in deposits. In our view, radio makes information more correlated, therefore this finding also suggests that the higher the correlation in information, the more likely are massive withdrawals and bank runs in times of financial distress.

Regarding learning about the share of previous choices in the population, [21] is a closely related paper. In his model, individuals choose in a one-shot game between binary actions: playing stock or bond. He assumes that an increase in the fraction of the individuals playing stock increases the payoff related to that choice. Payoffs also depend on the aggregate state of the world and on idiosyncratic shocks. He shows—among others—that if only a sample of previous actions is observed, then multiple outcomes may arise and herds may form. [21] studies only the case of random sampling, he does not deal with the issue of correlated samples.

Note that this study is not about social learning in which people want to learn the quality of a product or in our case the bank. The model makes it clear in the next section, that the bank is known to work properly, there is no uncertainty about the fundamentals. Thus, if subsequent depositors withdraw in masses, then it is not the consequence of inferring that the bank is bad. In this sense, we do not have a herding model or a global game in which depositors receive noisy private signals about the quality of the bank. Note that [22] studies herding in financial intermediation and she assumes that depositors observe some previous actions. Her focus is on the signal extraction problem of depositors about the bank’s assets and she assumes away bank runs resulting from coordination failure. Depositors use the information about previous withdrawals to infer the quality of the assets. If their belief about this quality is low, then they withdraw. Since more withdrawals suggest that previous depositors inferred that the assets are not performing well, more withdrawals are more likely to get a depositor to withdraw as well. The idea that observing more withdrawals may start a withdrawal wave is common in Gu’s and our paper. However, in our paper the fundamentals are good and we focus on the possibility of coordination failure. The study rather can be seen as a special coordination game with two types of players in which the players observe a set of previous decisions and decide sequentially. Despite the obvious differences, correlation has been found an important factor in social learning as well. [23] show that too much correlation in observations in social learning may lead to suboptimal decisions.

Finally, our setup resembles the situation of epidemic diffusion on social networks where a disease or some behavior spreads along the links of a network after an initial set of agents has adopted it (see chapters 3 and 4 in [24] for an extensive review and [25] for an economic application). In our case, a sizable fraction of the population adopts the behavior of deposit withdrawal and the question is whether this behavior becomes dominant in the long run if depositors observe a sample of previous decisions and use a threshold rule to decide whether to adopt. Since we compare close knit-communities and random sampling, those papers are especially relevant to us that consider diffusion on small-world networks ([19]). Here the conclusion on the impact of network structure on the spread of behavior largely depends on the nature of the process in question. Regarding the automatic spread of diseases, random links that connect communities are found to be crucial for the diffusion, in consequence, the spread reaches more nodes and takes over the network in shorter time in random networks (see e.g. [26] and [27]). In contrast, for economically relevant phenomena (such as innovation or coordination), where adoption follows utility maximization and depends on the number of neighbors who also adopt, the clustering of links matters. If links are clustered and nodes are embedded in a smaller community, the behavior can establish itself first in the community of the early adopter and then it spreads to other parts of the network (see this phenomenon for cooperation in [28], the diffusion of knowledge in [29], and coordination in [30] and [31]). Our paper falls in the latter category since it presents a special version of the coordination game. Our conclusions also resemble that literature: we find that the withdrawal behavior is adopted by (almost) everybody in the long-run when observations overlap which happens in close knit-communities.

## 3 The model

We present a framework based on the canonical [15] model with two types of depositors to which we add sequential decision making and sampling. The optimal contract in this setting is such that if only those depositors withdraw who really need liquidity, then those who leave their funds deposited receive more money than those who withdraw. However, the more depositors withdraw, the less money the bank has for future payments. Depositors form a belief about the total number of withdrawals based on what they observe in their sample of previous choices following the law of small numbers and then decide whether to withdraw or keep the money in the bank.

### 3.1 A modified Diamond-Dybvig model

There are infinite depositors who form a bank and deposit their unit endowment there at *T* = 0. The bank invests the deposits in a safe technology which pays unit gross return after each endowment liquidated at *T* = 1 and *R* > 1 after each endowment liquidated at *T* = 2. The long-term return, *R*, is constant. Therefore, the bank is fundamentally in good conditions and there is no uncertainty in this regard. At the beginning of *T* = 1 a share 0 < *π* < 1 of the depositors is hit by a liquidity shock and becomes impatient, valuing only consumption in period 1 The rest is of the patient type who enjoy consumption in period 1 and 2. Preference types are not publicly observable and there is no aggregate uncertainty regarding the liquidity preference of the depositors.

At period 0 depositors form a bank by pooling their resources, so that they can share the risk of becoming impatient. Intuitively, the bank will liquidate at no cost some long-term investment in period 1 to pay a relatively high (that is, higher than their endowment) consumption to depositors who turn out to be impatient, while patient depositors may enjoy an even higher consumption in period 2. The bank offers this liquidity insurance through a simple deposit contract which is subject to a sequential service constraint. Hence, the bank commits to pay a fixed consumption (*c*_1_) to those who withdraw in period 1 (unless it runs out of funds) and a contingent payment in period 2 to those who keep deposited their money. The sequential service constraint means that the bank must pay *c*_1_ as a depositor claims her funds back in period 1. The depositors who keep the money deposited receive in period 2 a pro-rata share of the matured assets which have not been withdrawn during period 1. As usual in the literature ([32]), in period 1, depositors are isolated and no trade can occur among them.

The timing of events is as follows. At period 0 each depositor deposits in the bank her endowment which is invested in the technology. At the beginning of period 1 depositors learn their type privately and nature assigns a position in the line to each depositor. Depositors decide according to this exogenously determined sequence and we suppose that depositors do not know their position in the line and they assign a uniform probability of being at any possible position. This assumption follows [21]. Introducing (exact or approximate) knowledge about the position would complicate the analysis. Moreover, it seems rather unrealistic to assume that depositors have an accurate view about how many other depositors have already made a decision.

Assume that before decision depositors observe a sample of previous actions which they use to form beliefs about the share of depositors who choose to withdraw. Based on these beliefs, they choose either to withdraw their funds from the bank or keep it deposited (that we also call waiting). We denote withdrawal as action zero (*a*_*i*_ = 0) and keeping the money deposited as action 1 (*a*_*i*_ = 1).

Denote by (*c*_1_, *c*_2_) the consumption bundle of a depositor in the two periods. Consider the following utility function

$$\begin{array}{c} u\left(c_{1},c_{2},\theta_{i}\right)=u\left(c_{1}+\theta_{i}c_{2}\right), \end{array}$$

where *θ*_*i*_ is a binomial random variable with support {0,1}. After realization of the needs, if *θ*_*i*_ = 0, then depositor *i* is impatient caring only about consumption in period 1, *θ*_*i*_ = 1 represents a patient depositor. The utility function, *u*(.) is twice continuously differentiable, increasing, strictly concave, satisfies the Inada conditions and the relative risk-aversion coefficient −*cu*′′(*c*)/*u*′(*c*) > 1 for every *c*.

If types were publicly observable in period 1, then the bank could calculate the optimal allocation based on types and independently of the position in the line. Denote by $c_{T}^{\theta }$ the consumption of type *θ* in period *T*. Then the optimization problem takes the following form:

$$\begin{array}{l} \text{max }\pi u(c_{1}^{0}+c_{2}^{0})+(1-\pi )u(c_{1}^{1}+c_{2}^{1})\\ s.t.\\ \;\;\;\;(1)\;c_{2}^{0}=c_{1}^{1}=0\\ \;\;\;\;(2)\;\pi c_{1}^{1}+[(1-\pi )c_{2}^{2}/R]=1 \end{array}$$ (1)

The first restriction requires that impatient depositors consume in period 1 and patient depositors in period 2. This is optimal, because by consuming in period 2 patient depositors earn the return (*R* > 1). The second restriction is the resource constraint. For simplicity, we will denote $c_{1}^{1*}$ by $c_{1}^{*}$ and $c_{2}^{2*}$ by $c_{2}^{*}$. The solution to this problem is characterized by

$$\begin{array}{c} u^{\prime }\left(c_{1}^{*}\right)=Ru^{\prime }\left(c_{2}^{*}\right), \end{array}$$

which implies $R>c_{2}^{*}>c_{1}^{*}>1.$

The optimal allocation can be implemented by banks via a simple deposit contract. The depositors withdrawing in period 1 are given $c_{1}^{*}$, while those who keep their funds in the bank reap the benefit of the long-term investment and divide it equally among themselves. The consumption of those who wait depends on the mass of withdrawals in period 1 (*ω*) and can be expressed in the following way:

$$\begin{array}{c} c_{2}\left(\omega \right)=\left\{\begin{array}{c} \max\left\{0,\frac{R(1-\omega c_{1}^{*})}{1-\omega }\right\}\;\text{if}\;1-\omega >0\\ 0\;\text{if}\;1-\omega =0 \end{array}\right., \end{array}$$ (2)

If *ω* = *π*, then $c_{2}\left(\omega \right)=c_{2}^{*}$. Nevertheless, if *ω* is high enough, then withdrawing in period 1 is a better option for a patient depositor, than keeping the money deposited (provided the bank has money left to pay). There is a threshold value such that if the number of those who have withdrawn is over this threshold, then the period-2 consumption will be less than $c_{1}^{*}$.

**Lemma 1** *There exists a* $\pi \leq \bar{\omega }<1$ *such that*

$$\begin{array}{c} c_{2}\left(\omega \right)<c_{1}^{*}\;\text{for}\;\text{any}\;\bar{\omega }<\omega \\ \text{and}\\ c_{1}^{*}\leq c_{2}\left(\omega \right)\;\text{for}\;\text{any}\;\bar{\omega }\geq \omega . \end{array}$$ (3)

**Proof.** The bank cannot pay in period 1 to all depositors $c_{1}^{*}>1$, since depositors have a unit endowment and gross return upon withdrawing in the first period is 1. Hence, for any $\frac{1}{c_{1}^{*}}\leq \omega ,$ *c*_2_(*ω*) = 0. On the other hand, $c_{2}^{*}=c_{2}\left(\omega \right)$ for *ω* = *π* and $0<\frac{\partial c_{2}\left(\omega \right)}{\partial \omega }$ for any $\frac{1}{c_{1}^{*}}<\omega <\pi$, so by continuity of *c*_2_(*ω*) for $\frac{R(1-\omega c_{1}^{*})}{1-\omega }>0$ there is a unique $\bar{\omega }$ such that for any $\omega \leq \bar{\omega }$ we have $c_{1}^{*}\leq c_{2}\left(\omega \right)$, whereas for any $\bar{\omega }<\omega$ we have $c_{2}\left(\omega \right)<c_{1}^{*}.$

**Lemma 2** *Assume that the utility function exhibits constant relative risk aversion (CRRA) and takes the following standard from:*

$$\begin{array}{c} u\left(c_{i}\right)=\frac{c_{i}^{1-\delta }}{1-\delta }, \end{array}$$

*and* *δ* > 1, *R* > 1. *In this case the optimal allocation is the following:* $c_{1}^{*}=\frac{1}{\left(1-\pi \right)R^{\frac{1-\delta }{\delta }}+\pi }$, $c_{2}^{*}=\frac{R^{\frac{1}{\delta }}}{\left(1-\pi \right)R^{\frac{1-\delta }{\delta }}+\pi }$. *The threshold value* $\bar{\omega }$ *is given by* $\bar{\omega }=\frac{R-c_{1}^{*}}{c_{1}^{*}\left(R-1\right)}$. *We have the following comparative statics results:* $\frac{\partial \bar{\omega }}{\partial \pi }>0$, $\frac{\partial \bar{\omega }}{\partial \delta }<0$, *and* $\frac{\partial \bar{\omega }}{\partial R}<0$.

**Proof.** Since *δ* > 1, $\frac{1-\delta }{\delta }<0$ and given *R* > 1 we have that $R^{\frac{1-\delta }{\delta }}<1$. As a consequence, $\frac{\partial c_{1}^{*}}{\partial \pi }<0$. It is straightforward to show that $\frac{\partial \bar{\omega }}{\partial c_{1}^{*}}<0$, so $\frac{\partial \bar{\omega }}{\partial \pi }=\frac{\partial \bar{\omega }}{\partial c_{1}^{*}}\frac{\partial c_{1}^{*}}{\partial \pi }>0$. Similarly, $\frac{\partial c_{1}^{*}}{\partial \delta }>0$, therefore $\frac{\partial \bar{\omega }}{\partial \delta }=\frac{\partial \bar{\omega }}{\partial c_{1}^{*}}\frac{\partial c_{1}^{*}}{\partial \delta }<0$.

Regarding $\frac{\partial \bar{\omega }}{\partial R}$, we can compute it as $\frac{\partial \bar{\omega }}{\partial R}=\frac{\left(\pi -1\right)(R^{1/\delta }\left(\left(\delta -1\right)R+1\right)-\delta R)}{\delta {\left(R-1\right)}^{2}R}$. The sign of this derivative depends on the sign of *f*(*R*, *δ*)≡(*R*^1/*δ*^((*δ* − 1)*R* + 1) − *δR*). Some properties of *f*(*R*, *δ*): $\frac{\partial^{2}f\left(R,\delta \right)}{\partial^{2}R}=\frac{\left(\delta -1\right)R^{\frac{1}{\delta }-2}\left(\delta R+R-1\right)}{\delta^{2}}>0$ (given *R* > 1 and *δ* > 1), and if *R* = 1, $\frac{\partial f(R,\delta )}{\partial R}=0$, therefore $\frac{\partial f(R,\delta )}{\partial R}>0$ if *R* > 1. Further, *f*(1, *δ*) = 0, therefore *f*(*R*, *δ*)>0 if *R* > 1. From there we conclude that $\frac{\partial \bar{\omega }}{\partial R}<0$ if *R* > 1.

Patient depositors increase their optimal withdrawal threshold $\bar{\omega }$ when there are more impatient depositors in the economy since they are aware that more withdrawals are due to impatient and not patient depositors. The derivative of the threshold with respect to the relative risk aversion coefficient is negative ($\frac{\partial \bar{\omega }}{\partial \delta }<0$), so the more risk-averse are the depositors, the more they want to smooth the consumption. Consequently, higher risk aversion implies a smaller difference between $c_{1}^{*}$ and *c*_2_, so the threshold decreases.

As for $\frac{\partial \bar{\omega }}{\partial R}$, an increase in the return has a double effect. On the one hand, it increases the consumption given to those who withdraw in the first period ($c_{1}^{*}$) which—ceteris paribus—lowers the threshold ($\frac{\partial \bar{\omega }}{\partial c_{1}^{*}}<0$). On the other hand, a higher return increases also the period-2 consumption which has a positive effect on the threshold ($\frac{\partial \bar{\omega }}{\partial R}>0$). When *δ* > 1, the first effect dominates the second. This condition is a classic assumption in papers about bank runs (see for instance [15]).

We chose a utility function often used in the literature (see for instance [33] or [34]) to find in a reasonable way the threshold that determines if a depositor runs or not. Note that without imposing a utility function it is not obvious how to find the threshold in a consistent way. Clearly, the threshold depends on a host of factors. For instance, the higher is the share of impatient depositors, the higher should be the threshold: a threshold of 50% makes sense when the share of impatient is at most 50%, but does not seem reasonable if the share of impatient depositors is above 50%. Similarly, the more risk averse are depositors, the more worried they are about receiving nothing if they do not withdraw, leading to a lower threshold. All these considerations can be taken into account in a consistent way using the standard utility function put forward in the literature.

#### Sequential decisions and samples

Our setup differs from [15] in two points: depositors decide consecutively according to an exogenously predetermined sequence, and before making a decision they use a sample of previous choices to form beliefs about the share of depositors who withdraw in the first period.

Concerning the size of the sample, for simplicity we focus on the case where each depositor observes a sample of the same size, *N*. We suppose that this size is reasonably large. Our results are not driven by excessively small sample size, e.g. observing one or two previous actions. However, the size does not allow to draw a precise conclusion about the share of withdrawals in the whole population and—as it will be seen later—it affects the probability of bank runs. How do depositors decide at the beginning of the sequence? They do not have enough choices to observe. For simplicity, we assume that the first *m* (*N* ≤ *m*) depositors decide according to their type, that is impatient depositors withdraw, while patient ones keep their money deposited. This assumption allows us to study whether a bank run can emerge from a situation where everything goes as normal and the bank does not receive any shock in the expected returns. We consider theoretically two particular cases. One is the random sampling case in which depositor *i* is equally likely to observe any of her predecessors. The other case has recent predecessors oversampled in a special way. In this case—which we call overlapping sampling—only the last *N* depositors’ actions are observed. This assumption captures, in an extreme format, the idea that recent choices are more likely to be observed. Also, it can be argued that samples may overlap, for example, because individuals belong to the same close-knit community, or due to clustering, as already mentioned. We also consider the intermediate cases where samples are partially random and partially overlapping.

Depositors use the observed sample to form beliefs about the population share of individuals who withdraw their money from the bank in the first period. To this end, they look at the relative shares of different choices in their sample. Following the behavioral economics literature on the law of small numbers, we assume that a patient depositor believes that the sample is representative and informative of the whole population. Note that impatient depositors always withdraw, so we focus on the decision of patient depositors. For example, if she observes that 60% of her sample withdraws their money from the bank, then she makes some inference based on this information about the share of withdrawals by the end of period 1.

We denote by $\hat{\omega_{i}}$ the share of withdrawals in depositor *i*’s sample. To make a decision, depositors compare $\hat{\omega_{i}}$ to the theoretical threshold value $\bar{\omega }$ defined by Lemma 1. The decision rule can be summarized as:

$$\begin{array}{c} a_{i}\left(N,\hat{\omega_{i}}\right)=\left\{\begin{array}{c} 1\;\text{if}\;\hat{\omega_{i}}\leq \bar{\omega }\\ 0\;\text{if}\;\hat{\omega_{i}}>\bar{\omega } \end{array}\right., \end{array}$$ (4)

where decision *a*_*i*_ = 1 denotes keeping the money deposited, while *a*_*i*_ = 0 is withdrawal. If the share of withdrawals in her sample is larger than $\bar{\omega }$, then a patient depositor withdraws. Otherwise, she keeps the money deposited.

A depositor observing a relatively large number of withdrawals believes that what she observes is representative of the proportion of withdrawals at the end of the period. Therefore, it is optimal for her to withdraw her funds from the bank. The rationality of the proposed decision rule may be questioned on the following basis. Our decision rule does not take into account the effect the decision has on the choices of subsequent depositors. This effect is based on the probability that their decision will be sampled by subsequent depositors. Since the samples determine the decision of those depositors, the effect of leaving the money with the bank may be important. The effect is larger for depositors at the beginning of the line and it also depends on the sampling mechanism. [21] shows in an investment setup that with infinite players inferences about the position are irrelevant for strategies and players can ignore the effects of their own decision on the behavior of others. This lends some support to our modeling choice.

We are interested in whether bank run emerges in our setup or not. A natural way to study this question is to see whether a massive withdrawal wave arises. We define bank run in this paper as a situation in which most depositors withdraw in the long run.

**Definition 1** *There is a bank run if the share of waiting depositors* (*k*) *becomes small in the long run:* k* < *ϵ* *where* *ϵ* *is an arbitrarily small positive number*.

Hence, if the share of those who do not withdraw converges to zero, then we consider it as a bank run. In the theoretical results (section 3.2 and 3.3) we consider the case when this share converges to zero, while in the simulations (section 4) we define a bank run when this share is less than 3% among the last 20000 depositors. Note that once there have been depositors who left their money in the bank, it has some probability that a patient depositor happens to observe the decisions of those depositors even though there has been a lot of patient depositors withdrawing in front of her. To account for this possibility, we allow for the case that a small fraction of patient depositors keep their funds deposited.

### 3.2 Overlapping samples

In this subsection, we study the probability of bank runs when depositors observe the last decisions. We introduce the following notation. Let $\varphi_{i}={\hat{\omega }}_{i}N$ and $\tau =\bar{\omega }N$, that is, *φ*_*i*_ denotes the *number* of depositors who withdraw in *i*’s sample and *τ* the threshold *number* of withdrawals in the sample that makes *i* to withdraw.

First, let us consider how the number of withdrawals evolves with overlapping samples. The number of withdrawals in subsequent samples is

$$\begin{array}{c} \varphi_{i}=\varphi_{i-1}+\left(a_{i-(N+1)}-a_{i-1}\right). \end{array}$$ (5)

where *a*_*i*−(*N*+1)_ is the choice of depositor *i* − (*N* + 1). The formula just says that the change in the number of withdrawals in subsequent samples depends on the decisions which are different in the samples. These are the decisions of depositor *i* − (*N* + 1) and depositor *i* − 1. The first one is in the sample of depositor *i* − 1, but not in the sample of depositor *i*, and with the latter one it is the other way around. Hence, the number of withdrawals can differ at most in one unit.

Given any possible threshold *τ*, it is easy to see that if there has not been any withdrawing patient depositor yet, then the only event that makes a patient depositor to withdraw is to have in her sample more than *τ* impatient depositors. If *φ*_*i*−1_ ≥ *τ* + 1, then according to the threshold rule *a*_*i*−1_ = 0, so depositor *i* − 1 withdraws. Notice that if *a*_*i*−1_ = 0, then *φ*_*i*−1_ ≤ *φ*_*i*_, because 0 ≤ *a*_*i*−(*N*+1)_−*a*_*i*−1_. This, in turn, implies that *φ*_*i*_ ≥ *τ* + 1, so depositor *i* withdraws as well. By continuing along these lines, we find that once there is a sample such that *φ*_*i*_ ≥ *τ* + 1, all subsequent depositors will observe a sample with a number of withdrawals which makes patient depositors to withdraw. Since impatient depositors withdraw always, a sample with more than *τ* withdrawals sets off a bank run.

**Lemma 3** *Given the threshold strategy, if* *φ*_*i*_ ≥ *τ* + 1 *for depositor* *i*, *then for any depositor* *i* ≤ *j* *we have that* *φ*_*j*_ ≥ *τ* + 1.

**Proof.** If *φ*_*i*_ ≥ *τ* + 1, then *a*_*i*_ = 0, so *φ*_*i*+1_ ≥ *φ*_*i*_. If depositor *i* is impatient, then she withdraws, as does a patient depositor *i*, since *φ*_*i*+1_ ≥ *φ*_*i*_ ≥ *τ* + 1. By the same arguments, any subsequent depositor will observe a number of withdrawal larger than *τ*.

Let us consider what happens if we add to this process the proposed decision rule.

**Corollary 1** *A bank run starts with the first patient depositor who has at least* *τ* + 1 *impatient depositors in her sample*.

**Proof.** It follows from the definition of bank run and the previous lemma.

If no patient depositor has withdrawn yet, then the number of withdrawals in the last *N* observations follows a binomial distribution with parameters *N* and *π*. The lower bound on the probability that a bank run starts within the next *N* depositors is given by

∑x=τ+1NNxπx(1-π)N-x>0, (6)

that is the probability that in the sample there are at least *τ* + 1 impatient depositors.

**Proposition 1** *With overlapping samples and the proposed decision rule, the probability of bank run is 1 and it is independent of* *τ*.

**Proof.** The proposition can be proven using the second Borel-Cantelli lemma which asserts that if the events *E*_*i*_ are independent and the sum of the probabilities of the *E*_*i*_ diverges to infinity, then the probability that infinitely many of them occur is 1.

Decompose the sequence of depositors into disjoint blocks consisting of *N* subsequent depositors. Denote by *E*_*κ*_ the event that at least *τ* + 1 impatient depositors appear in the *κ*^*th*^ block. Since types are distributed independently across depositors and blocks are disjoint, events *E*_*κ*_ are also independent. The probability to have at least *τ* + 1 impatient depositors in block *κ* is

Pr(Eκ)=∑x=τ+1NNxπx(1-π)N-x>0. (7)

Then $\sum_{\kappa =1}^{\infty }\mathrm{Pr}\left(E_{\kappa }\right)=\infty$, so the probability of having this event infinitely many times is one. For our purposes it is enough to know that it happens at least once and afterwards according to the previous corollary all depositors will withdraw.

#### Discussion

Notice that the results do not change even if patient depositors adjust for the possibility of observing just a sample with too many impatient depositors. The probability of such an event is given by Eq (6). This is the *p-value* of having at least *τ*+1 impatient depositors in the sample. Depositors could adjust the threshold (that is, they can choose a larger *τ*) in such a way that this *p-value* be made very small. It means that depositors want to minimize the error of withdrawing when no run is underway. The flipside of the argument is that it may increase the error of not withdrawing when a run is underway. Hence, the optimal extent of adjustment should also take into account the probability of not withdrawing when a run is underway and the loss of doing so. The intuition behind this idea is that if somebody observes a sample whose *p-value* is very low, then she has good reasons to believe that it is due to the fact that patient depositors have been withdrawing from the bank. It suggests that a bank run is really underway, so the best she can do is to withdraw as well. This kind of adjustment does not help, because as long as *τ* < *N*, in an infinite sequence almost surely there will be a sample consisting of only impatient depositors. As a consequence, a bank run starts.

Consider a patient depositor who observes a sample with *τ* < *φ*_*i*_ < *N* withdrawals. When observing that at least one depositor has left the money in the bank, the depositor knows that there was a patient depositor before her who—based on her sample—decided to not withdraw. It may suggest that the subsequent depositors who have withdrawn, did it due to being impatient. These considerations are in line with increasing the threshold. In the extreme case, the decision rule may prescribe to keep the money deposited if there is at least one depositor in the sample who did so. We have seen that our result is independent of the threshold, so these arguments do not change the conclusions. Notice also that given an infinite sequence of depositors increasing the sample size does not change the conclusions either.

Note that Proposition 1 extends to the case when depositors differ in their threshold value. This may be the case, for example, when depositors differ in the degree of risk aversion as the threshold $\bar{\omega }$ (and *τ*) depends on the parameter of relative risk aversion (see Lemma 2). Suppose that the threshold *τ*_*i*_ is distributed on the interval [0, *N*) such that the maximum value is *τ*_*max*_ < *N*. In this case a bank run starts when a depositor with threshold *τ*_*max*_ has *τ*_*max*_ + 1 impatient depositors in her sample. This event has positive probability and therefore surely happens in an infinite sequence. All subsequent patient depositors will also withdraw since their threshold is less or equal to *τ*_*max*_.

The result is discouraging, because it tells that bank runs resulting from coordination failures when the last decisions are observable are pervasive. We predict a unique outcome which is just the consequence of chance that determines the type vector according to which depositors decide. The assumption of countably infinite individuals is instrumental to obtain that a bank run arises with certainty. If we consider a model with finite number of depositors, then the result may change, depending on the size of the population. We run simulations with a finite population of depositors and change the population size between 10^3^ and 10^7^. The program code used to make all simulations in this paper is available in the Supporting Information (S1 File). Following Example 1, we use the CRRA utility function and endogenously compute the threshold $\bar{\omega }$. We change the sample size between 10 and 60, the second-period investment return (R) is set to 1.3, the relative risk aversion parameter to 2.5. We assume that the first 100 agents act according to their type (*m* = 100). We change the share of impatient depositors between 0 and 1. We run 300 simulations for each parameter setting. Table 1 shows the frequency of bank runs in these 300 simulations. Note that a bank run arises whenever at least one patient withdraws, this follows from Corollary 1, we therefore only need to check whether this happens. We can observe that the probability of bank run rises with the population size, 10^6^ or 10^7^ depositors are sufficient to ensure that bank runs occur with certainty for most parameter values. Larger populations increase the chance that there is at least one sample that triggers bank run. For smaller population sizes the probability of bank runs can still be one if the sample size is small or the share of impatient depositors is large. Both of these conditions increase the chances that a particular sample is observed in which the share of impatient depositors is larger than the threshold such that a bank run starts. Note that even if the probability of bank run is below one for smaller population sizes, our main result that increasing the share of random observations reduces the likelihood of bank runs carries through. We interpret this result as comparative statics for a given size of the population.

**Table 1:** Simulations for the overlapping case with different population sizes.

*N* = 10					
*π*/Population size	10^3^	10^4^	10^5^	10^6^	10^7^
0.1	0.363	0.997	1	1	1
0.3	1	1	1	1	1
0.5	1	1	1	1	1
0.7	1	1	1	1	1
0.9	1	1	1	1	1
*N* = 35					
*π*/Population size	10^3^	10^4^	10^5^	10^6^	10^7^
0.1	0	0	0.007	0.023	0.183
0.3	0.15	0.763	1	1	1
0.5	0.867	1	1	1	1
0.7	1	1	1	1	1
0.9	1	1	1	1	1
*N* = 60					
*π*/Population size	10^3^	10^4^	10^5^	10^6^	10^7^
0.1	0	0	0	0	0
0.3	0.004	0.033	0.253	0.923	1
0.5	0.133	0.783	1	1	1
0.7	0.87	1	1	1	1
0.9	1	1	1	1	1

The parameters are set as *R* = 1.3, *δ* = 2.5, *m* = 100. The share of impatient depositors *π* and the sample size *N* are changed as shown in the Table. 300 simulations are run for each parameter setting. The probability of bank run is computed as the percentage of simulation runs where a bank run occurred (out of 300 simulation runs). A bank run occurs in a given simulation run if at least one patient depositor withdraws which sets a cascade off.

In a somewhat similar vein, [35] studies myopic best response in an evolutionary banking setup. In his model with local interaction depositors observe the share of banks that suffered a bank run in the previous period (and not a sample of previous decisions). He finds that once a bank experiences a run, then possibly a panic ensues involving all banks experiencing a bank run. This is analogous to our result that once a patient depositor withdraws, all subsequent depositors withdraw. However, bank run is not necessary theoretically even if what subsequent depositors observe are similar. [17] show that if depositors decide sequentially and each of them observes *all* previous decisions, then bank run is not an equilibrium outcome. Therefore, it is not correlation in the observed previous choices *per se* that leads to Proposition 1, but correlation and observing only a sample.

### 3.3 Random samples

In this section, we study the case of random samples, the analysis in this section builds heavily on section 5 in [21] that provides a very clear way how to find equilibria in this kind of problems. We assume that each depositor observes any of the previous decisions with equal probability (and without replacement). If the share of depositors who decided to keep their money deposited up to *i* − 1 is *k*_*i*−1_, then the probability that depositor *i* will observe a sample of size *N* with exactly *φ*_*i*_ withdrawals is given by the hypergeometric distribution:

χi(φi∣i,N,ki-1)=(1-ki-1)(i-1)φiki-1(i-1)N-φii-1N. (8)

When the population size is large, the hypergeometric distribution can be approximated by the binomial distribution:

χi(φi∣i,N,ki-1)=Nφi(1-ki-1)φi(ki-1)N-φi (9)

We study the dynamics of the decisions if depositors follow the threshold decision rule. A patient depositor leaves the money in the bank when observing a sample with at most *τ* withdrawals, this happens with probability $\sum_{\varphi_{i}=0}^{\tau }\chi_{i}\left(\varphi_{i}|i,N,k_{i-1}\right)$.

The key issue is to find out whether *k*_*i*_ converges and if it is the case, then to which value. Based on Theorem 2.1 and Corollary 2.1 in [36] we know that our process converges almost surely to a limit variable. Moreover, Corollary 3.1 implies that this limit variable is a fixed point (note that in stochastic process theory the random case is a special case of a generalized Polya urn process. For more details see, for instance, [36] and [37]). If for a given threshold decision rule characterized by the threshold (*τ*) *k*_*i*_ converges to *k*, then *χ*_*i*_(*φ*_*i*_∣*i*, *N*, *k*_*i*−1_) converges to *χ*(*φ*∣*N*, *k*). Remember that if *k* converges to zero than it means that in the limit nobody keeps her money deposited, so there is a bank run. Therefore, if *k* tends to a positive number, then there is no bank run according to our definition.

Given the share of depositors who did not withdraw (*k*), we define the probability of observing a sample with a number of withdrawals over the threshold as

$$\begin{array}{c} e\left(\tau ,k\right)=\sum_{\varphi =\tau +1}^{N}\chi \left(\varphi |N,k\right). \end{array}$$ (10)

Note that 0 < *e*(*τ*, *k*)<1 for any *τ* ∈ (0, *N*). Consider depositor *i* and suppose that sufficiently many depositors have already decided. The share of depositors who decided to keep their money deposited up to her is *k*_*i*−1_. Then, the share of withdrawals is (1 − *k*_*i*−1_) and it equals the share of impatient depositors and the share of those patient depositors who happen to observe more than *τ* withdrawals. Formally,

$$\begin{array}{c} 1-k_{i-1}=\pi +\left(1-\pi \right)e\left(\tau ,k_{i-1}\right). \end{array}$$ (11)

As depositor *i* decides, the share of those depositors who did not withdraw changes to *k*_*i*_ and after each new decision this share varies again. The expression has a recursive structure, and after sufficient decisions the share of withdrawals converges to some 1 − *k*. If convergence occurs, then the following condition is met:

$$\begin{array}{c} 1-k=\pi +(1-\pi )e(\tau ,k). \end{array}$$ (12)

After some straightforward manipulation we get

k=(1-π)∑φ=0τNφ(1-k)φ(k)N-φ (13)

This condition means that the share of depositors who keep their money deposited is equal to the share of patient depositors who (i) are patient and (ii) observe less withdrawals than the threshold *τ*. Finding *k* is a fixed point problem. We are interested in stable crossings.

Notice that *k* = 0 is always a solution, because if the share of depositors who do not withdraw is zero, then all depositors withdraw independently of the threshold, so the proportion of depositors keeping their funds deposited is equal to zero. The question is whether there is a *k* > 0, implying that not all depositors withdraw in the long run. There is no bank run if and only if ∃*k* > 0 such that satisfies Eq (13).

Given the complexity of *e*(*τ*, *k*), we cannot assess simply by looking at Eq (13) if there is a positive solution to it. We analyze whether and for which parameter values a bank run occurs in two different ways that lead to the same results. First, we graphically show the solutions of Eq (13) and assess the impact of the parameters on the existence of a positive solution in *k* (indicating an outcome without bank runs). Second, we use simulation methods to verify the results obtained by the graphical solution.

Four parameters of the model determine the outcome: the investment return (*R*), the coefficient of relative risk aversion (*δ*), the share of impatient depositors (*π*), and the sample size (*N*). Note that *R* and *δ* influence only the decision threshold $\bar{\omega }$. We consider three scenarios for the values of these parameters: *Scenario 1:* *R* = 1.1, *δ* = 1.5; *Scenario 2:* *R* = 1.3, *δ* = 2.5; *Scenario 3:* *R* = 1.5, *δ* = 4. These scenarios cover the plausible ranges for the values of *R* and *δ*. We assume that the rate of investment return can be between 10 to 50 percent, a plausible range. The coefficient of relative risk aversion is estimated to be between 0 and 4 (see [38] and [39]), while in our case we need *δ* > 1 to guarantee that the consumption in the first period exceeds the endowment of the depositors, i.e. $c_{1}^{*}>1$. We assume that *δ* > 1, similarly to other studies about bank runs. The three scenarios of the parameter values correspond to high, mid-range and low values of the decision threshold, respectively (recall that the threshold $\bar{\omega }$ is decreasing in *δ* and *R*, see Lemma 2). Table 2 shows the threshold values $\bar{\omega }$ for these three scenarios and as the function of the fraction of impatient depositors (*π*) that we change between 0.1 and 0.9. We can see in the table that the threshold increases with *π* (as shown theoretically in Lemma 2). Note that *π* influences the occurrence of bank runs both through the threshold $\bar{\omega }$ and directly through Eq (13). Regarding the sample size *N*, our analysis covers the values between 10 and 210. Note that individuals may interact with more people on social media, however, there is evidence that active interactions are limited to only a small share of online contacts (see [40]). The sample size does not influence the decision threshold, it only appears in Eq (13).

**Table 2:** Decision threshold values ($\bar{\omega }$) for different parameter settings of *R*, δ and π.

	Scenario 1	Scenario 2	Scenario 3
*R*	1.1	1.3	1.5
*δ*	1.5	2.5	4
	Threshold values $\bar{\omega }$		
*π* = 0.1	0.690	0.432	0.292
*π* = 0.2	0.725	0.495	0.371
*π* = 0.3	0.759	0.558	0.449
*π* = 0.4	0.794	0.621	0.528
*π* = 0.5	0.828	0.684	0.607
*π* = 0.6	0.862	0.748	0.685
*π* = 0.7	0.897	0.811	0.764
*π* = 0.8	0.931	0.874	0.843
*π* = 0.9	0.966	0.937	0.921

$\bar{\omega }$ is computed using the expressions from Lemma 2 (where a CRRA utility function is assumed). These threshold values are used in the simulations and the numerical solutions of Eq (13).

**Fig 1:**
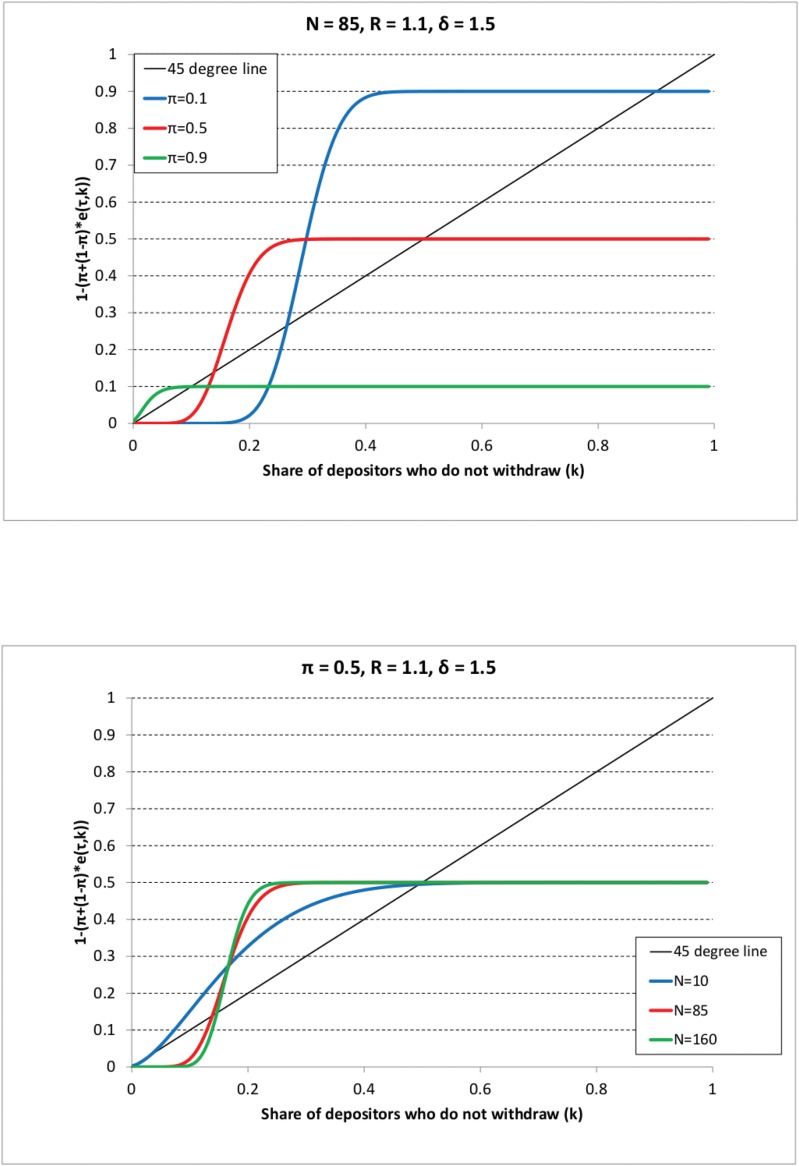
The long-run theoretical share of depositors who do not withdraw (*k*) in the case of random sampling and Scenario 1. The black line represents the left-hand side of Eq (13) (i.e. the 45-degree line), the colored lines represent the right-hand side of Eq (13) for different parameter values as shown in the legend. The long-run share of depositors who do not withdraw is given by the *largest* (rightmost) crossing point of the 45-degree line and a given colored line. The parameter values are as in Scenario 1 (*R* = 1.1, *δ* = 1.5). And on the first Panel: *N* = 85, *π* is varied as *π* = 0.1 (blue line), *π* = 0.5 (red line), *π* = 0.9 (green line). On the second Panel: *π* = 0.5, *N* is varied as *N* = 10 (blue line), *N* = 85 (red line), *N* = 160 (green line).

**Fig 2:**
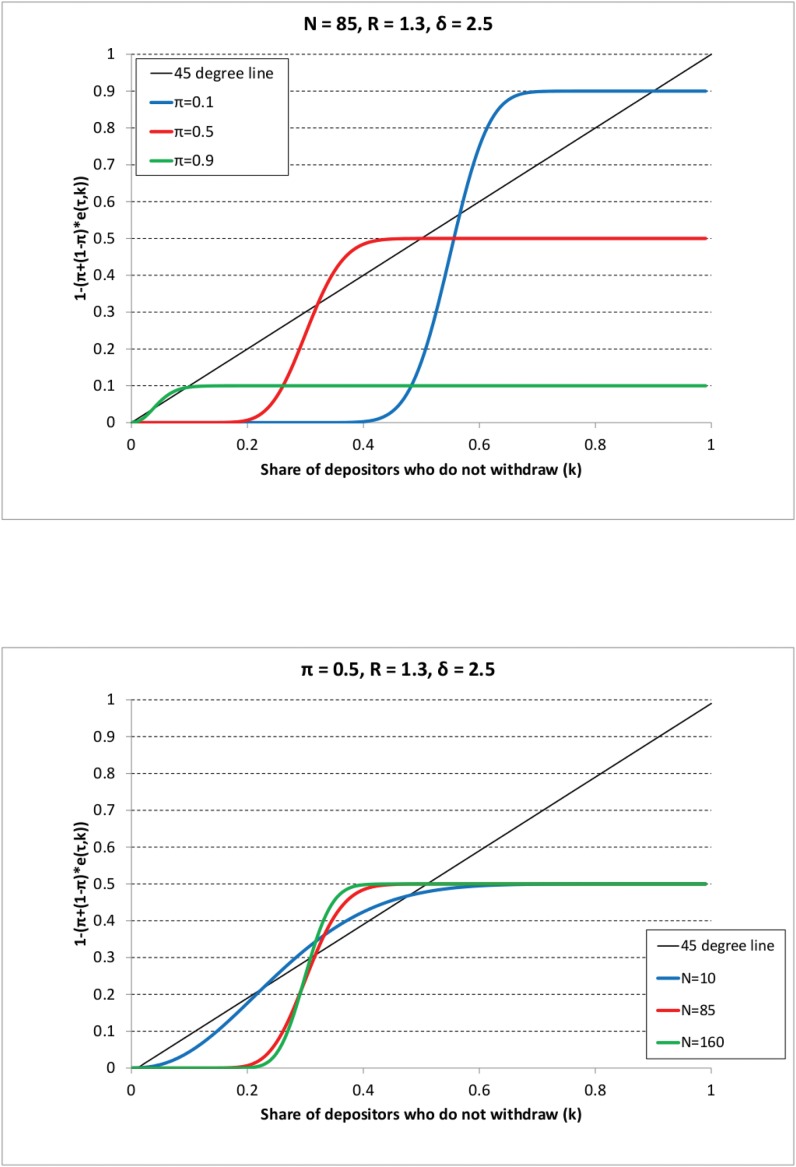
The long-run theoretical share of depositors who do not withdraw (*k*) in the case of random sampling and Scenario 2. The black line represents the left-hand side of Eq (13) (i.e. the 45-degree line), the colored lines represent the right-hand side of Eq (13) for different parameter values as shown in the legend. The long-run share of depositors who do not withdraw is given by the *largest* (rightmost) crossing point of the 45-degree line and a given colored line. The parameter values are as in Scenario 2 (*R* = 1.3, *δ* = 2.5). And on the first Panel: *N* = 85, *π* is varied as *π* = 0.1 (blue line), *π* = 0.5 (red line), *π* = 0.9 (green line). On the second Panel: *π* = 0.5, *N* is varied as *N* = 10 (blue line), *N* = 85 (red line), *N* = 160 (green line).

**Fig 3:**
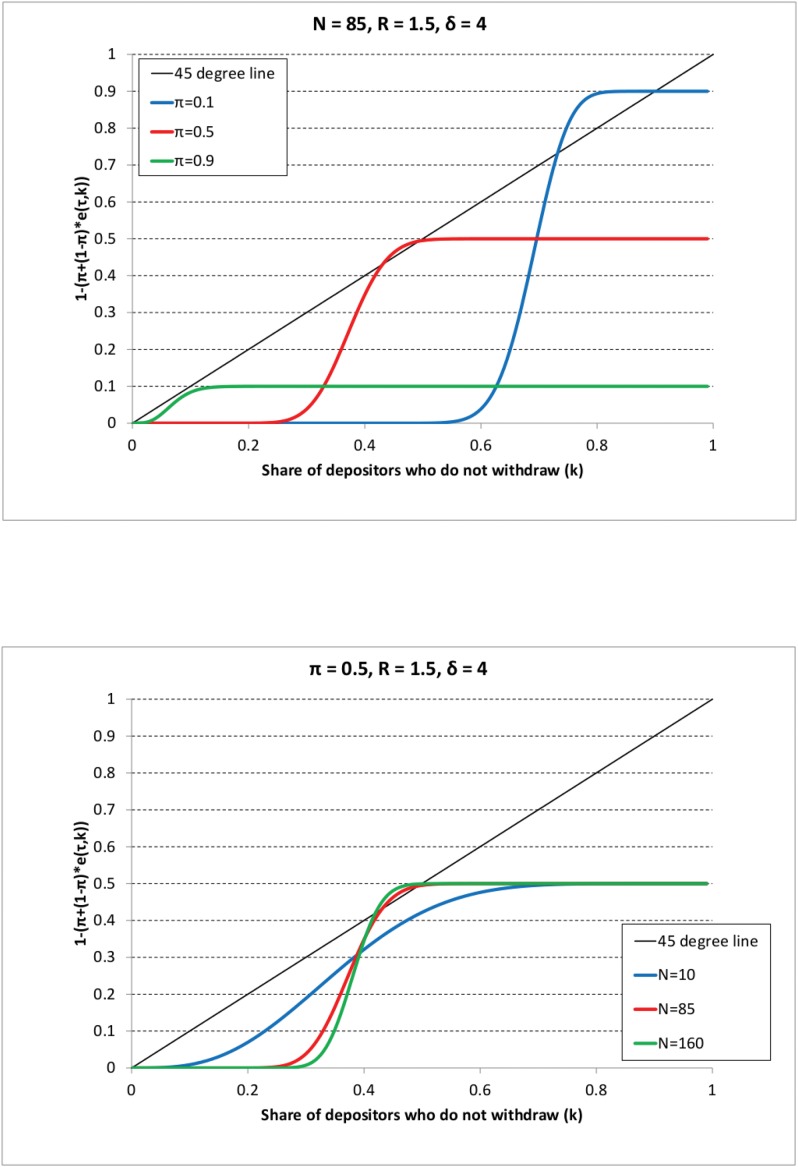
The long-run theoretical share of depositors who do not withdraw (*k*) in the case of random sampling and Scenario 3. The black line represents the left-hand side of Eq (13) (i.e. the 45-degree line), the colored lines represent the right-hand side of Eq (13) for different parameter values as shown in the legend. The long-run share of depositors who do not withdraw is given by the *largest* (rightmost) crossing point of the 45-degree line and a given colored line. The parameter values are as in Scenario 3 (*R* = 1.5, *π* = 4). And on the first Panel: *N* = 85, *π* is varied as *π* = 0.1 (blue line), *π* = 0.5 (red line), *π* = 0.9 (green line). On the second Panel: *π* = 0.5, *N* is varied as *N* = 10 (blue line), *N* = 85 (red line), *N* = 160 (green line).

Figs 1–3 show the different outcomes. On all graphs, the 45° line represents the left-hand side of Eq (13), whereas the colored curves represent the right-hand side of the same equation varying either the share of impatient depositors (*π*) or the sample size (*N*). Consider first Fig 1a that shows Scenario 1 where the decision threshold $\bar{\omega }$ is the highest (corresponding to *R* = 1.1 and *δ* = 1.5). For instance, following the blue line (representing the case with *π* = 0.1) from the right to the left it indicates that first 10% of depositors withdraw (corresponding to the impatient depositors), hence the share of those who do not withdraw (represented on the vertical axis) is 0.9. Recall that it is assumed that at the beginning of the line, agents decide according to their types, i.e. impatient withdraw, patient keep their money in the bank. As we go from the right to the left and *k* decreases, at some point the curve starts to decrease showing that if the share of previous withdrawals is high, then more and more patient depositors withdraw as well, so the share of those who do not withdraw becomes smaller. We are interested in the point where the curve first crosses the 45° line from the right as it represents the solution to the Eq (13). If it occurs for a k* > 0, then there is no bank run according to our definition. In other words, we concentrate on the largest stable crossing point in *k* only because even if there are other crossing points for smaller values of *k*, these points are not reached. This is because we start from the right on the graph where (*k* = 1 − *π*), so first only impatient depositors withdraw, then some patient ones may join in, but as we reach the first crossing that is a stable point, the process settles there. We can observe that bank runs never happen as in all cases we have a crossing point of the two sides of Eq (13) where the share of depositors keeping their money in the bank is positive, i.e. k* > 0. Fig 1 shows that if the decision threshold is large enough, due to low coefficient of relative risk aversion or low investment returns, bank runs never occur, independently of the sample size and the share of impatient depositors.

Considering Scenario 2 on Fig 2, the decision threshold is somewhat lower than in the previous case. The graphs indicate that bank runs do not occur for the parameter settings depicted on the graph.

In Scenario 3 (*R* = 1.5, *δ* = 4) the decision threshold is lower than in the two previous cases, withdrawal cascades have a higher chance to emerge. We can indeed observe on Fig 3 that if either the sample size is low (*N* = 10) or the share of impatient depositors is large (*π* = 0.9), the only crossing point is at *k* = 0, a bank run occurs. On the contrary, we obtain that there is no bank run if either the sample size is large enough or the share of impatient depositors is low.

The analysis provided by Eq (13) shows the average long-run share of depositors who keep their money in the bank. However, due to randomness in the initial conditions (that is, the actual share of patient depositors at the beginning of the sequence may vary) and in the sampling (e.g. patient depositors may happen to observe more impatient depositors), the actual dynamic process may not always converge to this average long-run outcome. Therefore, we use simulation methods to verify the previously obtained results. In the simulations, we consider a large population of 10^7^ depositors, because our results are obtained for an infinite sequence so we need many depositors. We compute the decision threshold following Lemma 2. We assume that the first 50000 depositors act according to their type (*m* = 50000), that is, impatient depositors withdraw, patient depositors keep their money in the bank. After these initial set of depositors, we start the process whereby patient depositors decide about withdrawal based on a random sample of size *N* and the threshold decision rule. We are interested in the long-run share of waitings (*k*), we measure it using the last 20000 depositors and record the percentage of depositors who keep their money in the bank. In line with Definition 1, we say that a bank run occurred if this percentage is below 3%. We have experimented with alternative thresholds (1%, 5%), the results remain unchanged, therefore we omit them from the paper. For each parameter setting, we carry out 100 simulation runs and assess the probability of bank runs based on how many times out of the 100 simulations a bank run happened. We tested whether this number of simulation runs gives robust results by running the model for several sets of 100 runs. We carried out this test for several parameter settings. The different simulation sets of 100 runs give exactly the same probability of bank runs, thus we concluded that it is sufficient to run 100 simulations per parameter setting.

Table 3 shows the simulation results for the different Scenarios described above. Most entries in the table are zeros and ones which means that the model predicts a unique outcome of the model for most parameter sets. This is in accordance with the previous graphical analysis. While in the original Diamond and Dybvig model with simultaneous decisions multiple equilibria prevail, our model predicts a single long-run equilibrium for both the random and the overlapping sampling structures. The few entries where the probability of bank run is between 0 and 1 indicate that in some cases the outcome is sensitive to the randomness in the initial conditions and the sampling process.

**Table 3:** The probability of bank run in the case of random sampling as computed from the simulations.

Scenario 1					
*N*	*π* = 0.1	*π* = 0.3	*π* = 0.5	*π* = 0.7	*π* = 0.9
10	0	0	0	0	0.9
35	0	0	0	0	0
60	0	0	0	0	0
85	0	0	0	0	0
110	0	0	0	0	0
135	0	0	0	0	0
160	0	0	0	0	0
185	0	0	0	0	0
210	0	0	0	0	0
Scenario 2					
*N*	*π* = 0.1	*π* = 0.3	*π* = 0.5	*π* = 0.7	*π* = 0.9
10	0	0	1	0	0.93
35	0	0	0	0	1
60	0	0	0	0	0.65
85	0	0	0	0	0
110	0	0	0	0	0
135	0	0	0	0	0
160	0	0	0	0	0
185	0	0	0	0	0
210	0	0	0	0	0
Scenario 3					
*N*	*π* = 0.1	*π* = 0.3	*π* = 0.5	*π* = 0.7	*π* = 0.9
10	1	1	1	1	0.88
35	0	1	1	1	1
60	0	0	1	1	1
85	0	0	0	1	1
110	0	0	0	1	1
135	0	0	0	1	1
160	0	0	0	1	1
185	0	0	0	0	1
210	0	0	0	0	1

The probability of bank run is computed as the percentage of simulation runs where a bank run occurred (out of 100 simulation runs). A bank run occurs in a given simulation run if less than 3% of the last 20000 depositors in the line keep their money in the bank. The population consists of 10^7^ depositors. The first panel shows the results for Scenario 1 (*R* = 1.1, *δ* = 1.5), the second panel for Scenario 2 (*R* = 1.3, *δ* = 2.5), the third panel for Scenario 3 (*R* = 1.5, *δ* = 4). The values of *N* and *π* are varied as shown in the first column and first row of each panel, respectively. The underlined entries can be directly compared to the outcomes represented on Figs 1–3.

Table 3 shows identical results to the previous graphical analysis. The underlined numbers in the table mark the cases that we also represented on Figs 1–3. The two kinds of analysis yields always the same results. For example, the first panel of Table 3 and Fig 1 show the outcome of the model for Scenario 1 where *R* = 1.1 and *δ* = 1.5. In all graphically represented cases there was no bank run, and the simulations also indicate that the probability of bank run is zero (see the underlined entries in Table 3). On the contrary, in Scenario 3 (*R* = 1.5, *δ* = 4) we obtained a run outcome for *N* = 10, *π* = 0.5 where the simulation also indicates that the probability of bank run is 1 (see the third panel of Table 3).

With respect to the impact of the parameters, Table 3 indicates that for Scenario 1, where the decision threshold is high due to the low values of *δ* and *R*, bank runs almost never emerge (except the case of *N* = 10 and *π* = 0.9). Comparing the entries across the panels Table 3, we can see that as the decision threshold decreases, the probability of bank runs changes from zero to one, but only for smaller values of the sample size (*N*) and larger values of the share of impatient depositors (*π*). Withdrawal cascade emerges in our model if many patient depositors observe sufficiently many impatient depositors and this accumulates over time. Intuitively, there is a higher chance for this accumulation if patient depositors need less observations of withdrawals to decide to withdraw. Table 3 also reveals that, holding everything else constant, the probability of bank runs increases with the share of impatient and decreases with the sample size, when the decision threshold is sufficiently low. If the share of impatient depositors rises, patient agents correct their decision threshold upwards (see Lemma 2). However, in some cases when the threshold is relatively low, this correction does not outweigh the direct effect of *π* that increases the likelihood of observing too many impatient depositors. Regarding the impact of the sample size, with larger samples there is a smaller chance that a patient depositor observes too many impatient depositors. Recall that the decision threshold is larger than the share of impatient depositors (see $\pi <\bar{\omega }$ from Lemma 1). Therefore if the sample size is large, the fraction of impatient depositors within the sample should be close to *π*, that is below the threshold, implying that patient depositors keep their money in the bank. These effects of *π* and *N* are only present for relatively lower thresholds (as in Scenario 2 and 3, see the second and third panels Table 3). When the threshold is large as in Scenario 1 (see the first panel of Table 3), the mentioned effects are not sufficient to change the probability of bank run from zero to one.

The following result summarizes our findings in this section:

**Result 1** *Considering random samples, we mostly observe a unique outcome of the model. This outcome may be either a bank run or a no-run outcome depending on the parameter values. The probability of bank run weakly increases with the share of impatient depositors* (*π*), *and weakly decreases with the sample size* (*N*) *and the decision threshold* ($\bar{\omega }$), *ceteris paribus*.

#### Discussion

Remember that the bank in our model is assumed to be fundamentally healthy, so bank runs are clearly suboptimal. However, they still occur in the random setting as well, but they are considerably less likely to happen than in the overlapping case. As indicated earlier, in real life good banks also suffer bank runs and in many occasions one of the driver of the bank run is that depositors observe other depositors rushing to the bank. Our results suggest that there is some room for preventing runs on good banks by making samples less correlated. More precisely, when massive withdrawals are observed by depositors (for instance through a TV broadcast showing long queues in front of a bank), then the bank or the authority responsible for financial stability should make also visible the decisions of depositors who do not withdraw (for example showing other branches of the bank with no queues or reporting on the stability of deposits in the bank). Diversifying the depositor pool and not focusing only on one special community may also beneficial to avoid large correlation in information across depositors.

The stochastic process implied by random samples is very different from that related to overlapping samples. Most importantly, observing a sample which results in a withdrawal due to the threshold decision rule does not entail that subsequent depositors also will observe a sample with a large number of withdrawals. Thus, the correlation across samples which led to bank runs in the overlapping case is considerably reduced. Nevertheless, random sampling *per se* does not eliminate bank runs. Our preferred interpretation of the previous results is that of comparative statics: less correlation leads to less bank runs. This comparative statics exercise is the focus of the next section.

Note that the results in the overlapping and random cases rely heavily on the assumption that depositors may get to know that other depositors keep their funds in the bank. If only withdrawals were observable and there were no way to know if somebody does not withdraw, then the samples in any case would consists only of withdrawals and our threshold rule would lead to bank runs always. We may also assume that a sample consists of withdrawals and non-withdrawals and the latter may imply either a decision to keep the funds deposited or no decision yet. This would augment the uncertainty about the information content of non-withdrawals. However, the line of reasoning applied before would still apply, possibly after adjusting the thresholds adequately. More non-withdrawals would be necessary to convince depositors that no bank run is underway, but the correlation across samples would still matter.

## 4 Intermediate cases

So far we considered two polar cases of sampling and found that while highly overlapping samples lead to bank runs with certainty in our setup, with random samples this needs not be the case. It is of interest to know what occurs between these two extremes. As we go from highly to less correlated sampling does the probability of bank runs change gradually or are there sharp jumps?

To investigate the role of the degree of correlation/randomness across subsequent samples, we change the degree of randomness, denoted by λ, between 0 (overlapping sample) and 1 (random sample). A depositor observes λ*N* randomly drawn previous decisions and (1 − λ)*N* directly preceding decisions in the line. Notice that for some parameter values, *λN* is not an integer number. In these cases we use the rounded values in the simulations. We study by simulations the frequency of bank runs for different parameter values. The simulation setup is similar to the simulations applied in the previous section. The population size is 10^7^, the first 50000 depositors act according to their type. We use the last 20000 depositors to measure the long-run share of withdrawals/waitings. We say that a bank run happened if the long-run share of waitings is below 3%. The decision threshold is computed according to the results in Lemma 2. For each parameter setting we run 100 simulations which proved to be sufficient to obtain a robust number for the probability of bank runs.

**Fig 4:**
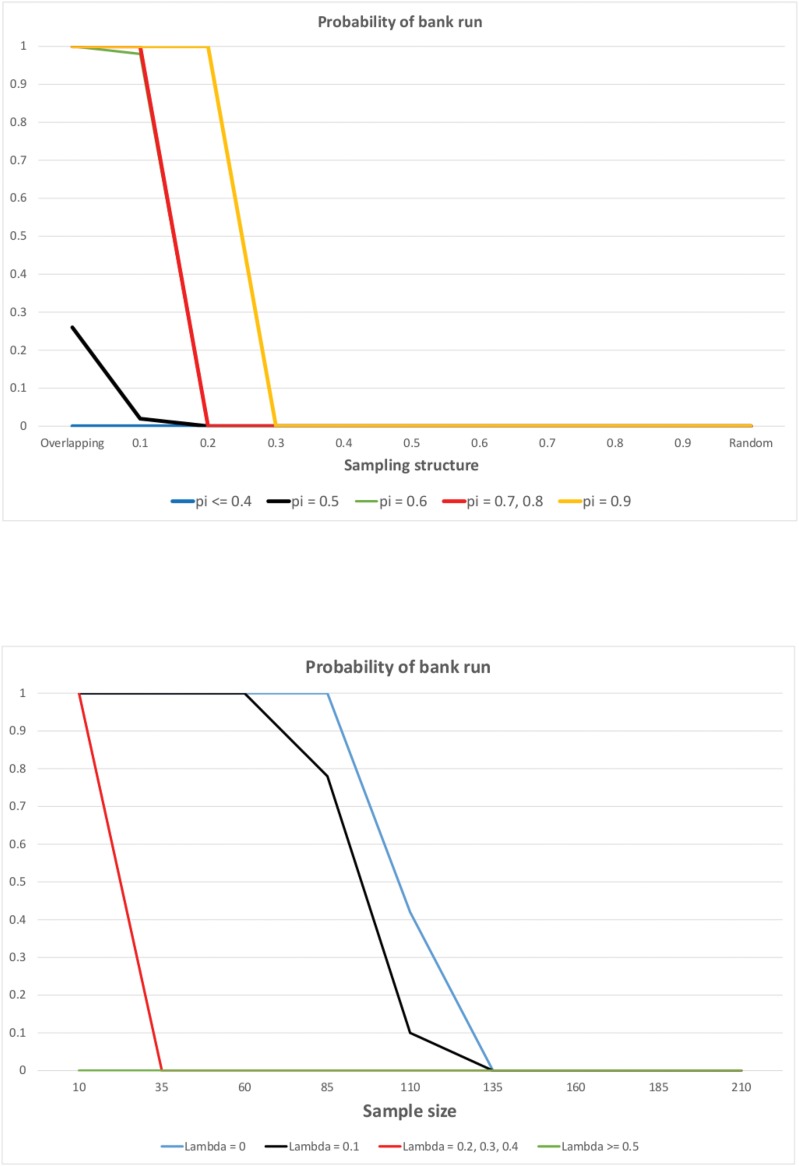
The probability of bank run as the function of the sampling structure (first panel) and the sample size (second panel), as computed from the simulations. Each depositor observes a random sample of size λ*N* and his (1 − λ)*N* immediate predecessors in the line. The probability of bank run (y-axis) is computed as the percentage of simulation runs where a bank run occurred (out of 100 simulation runs). A bank run occurs in a given simulation run if less than 3% of the last 20000 depositors in the line keep their money in the bank. The population consists of 10^7^ depositors. The parameters are set to *R* = 1.1, δ = 1.5 (as in Scenario 1). First panel: The x-axis represents λ going from 0 (overlapping case) to 1 (random case). *N* = 60 and *π* is varied between 0.1 and 0.9. Second panel: The x-axis represents the sample size *N* going from 10 to 210. *π* = 0.7 and the sampling structure λ is varied between 0 (overlapping case) and 1 (random case).

We change the degree of sample randomness λ between 0 and 1. The first panel of Fig 4 shows its impact on the probability of bank runs for Scenario 1 and setting the sample size to 60 (*N* = 60). We consider only Scenario 1 (*R* = 1.1, *δ* = 1.5) because in this case bank runs never occurred in the random case. Recall that in the overlapping case the probability of bank run is 1, so in Scenario 1 we can observe how the degree of randomness changes the probability of bank run going from 1 to 0. Conversely, if the probability of bank run is one in the random case as well, we do not expect any effect of λ. Note that if the share of impatient depositors is low (*π* ≤ 0.4), then bank runs do not occur even in the overlapping case. Proposition 1 stated that for infinite population and overlapping samples bank run occurs with probability 1. In the simulations, however, the population size is 10^7^ which seems to be too low to approximate an infinite population when the sample size is large. To trigger a bank run in the overlapping case, it is sufficient if one patient depositor observes sufficiently many impatient depositors just by chance. If the sample size is large enough and the share of impatient depositors is relatively small, this has a small probability, so we need a larger population such that this event of smaller probability indeed occurs. If we set the sample size to 10 (*N* = 10), then for any share of the impatient depositors the probability of bank run is 1 in the overlapping case. To study the effect of randomness, consider the cases with a relatively high share of impatient depositors (*π* > 0.4). We can observe on Fig 4 that for a given share of impatient depositors, as we go from highly correlated towards random samples, the probability of bank runs generally decreases. For smaller values of randomness (λ), the probability of bank run is one (in accordance with Proposition 1 for λ = 0) or at least above zero, and increasing λ leads to a sharp switch to a regime where the probability of bank run is zero. The transition from the regime with bank run to the regime without bank run for some values of *π* coincides, so the red line depicts this transition for *π* = 0.7 and 0.8. Interestingly, we can also observe that the higher is the share of impatient depositors, more randomness in the samples is needed for the transition to happen. For low values of *N*, the relationship between *π* and the randomness when transition occurs is not so neat in some cases.

The second panel of Fig 4 shows the impact of λ when the sample size is also changed (fixing the share of impatient depositors *π* = 0.7 and considering Scenario 1 again). We can observe that for λ ≥ 0.5 bank runs do not emerge for any value of the sample size. This is in accordance with the previous results that highly random samples are associated with no bank run. When λ < 0.5, bank runs do occur for smaller sample sizes, but not for larger ones. The sample size that is sufficiently large to eliminate bank runs depends on λ: the smaller is λ, the larger sample size is needed to bring the probability of bank run down to zero. In particular, for the pure overlapping case (λ = 0), the probability of bank run is zero for *N* ≥ 135. Again, the population size of 10^7^ seems to be too low to approximate an infinite population.

The following result summarizes the findings of this section:

**Result 2** *Bank runs are weakly less likely to occur, when the randomness in the sample increases. The probability of bank runs weakly decreases as the sample size becomes larger for any sampling structure represented by* λ.

**Fig 5:**
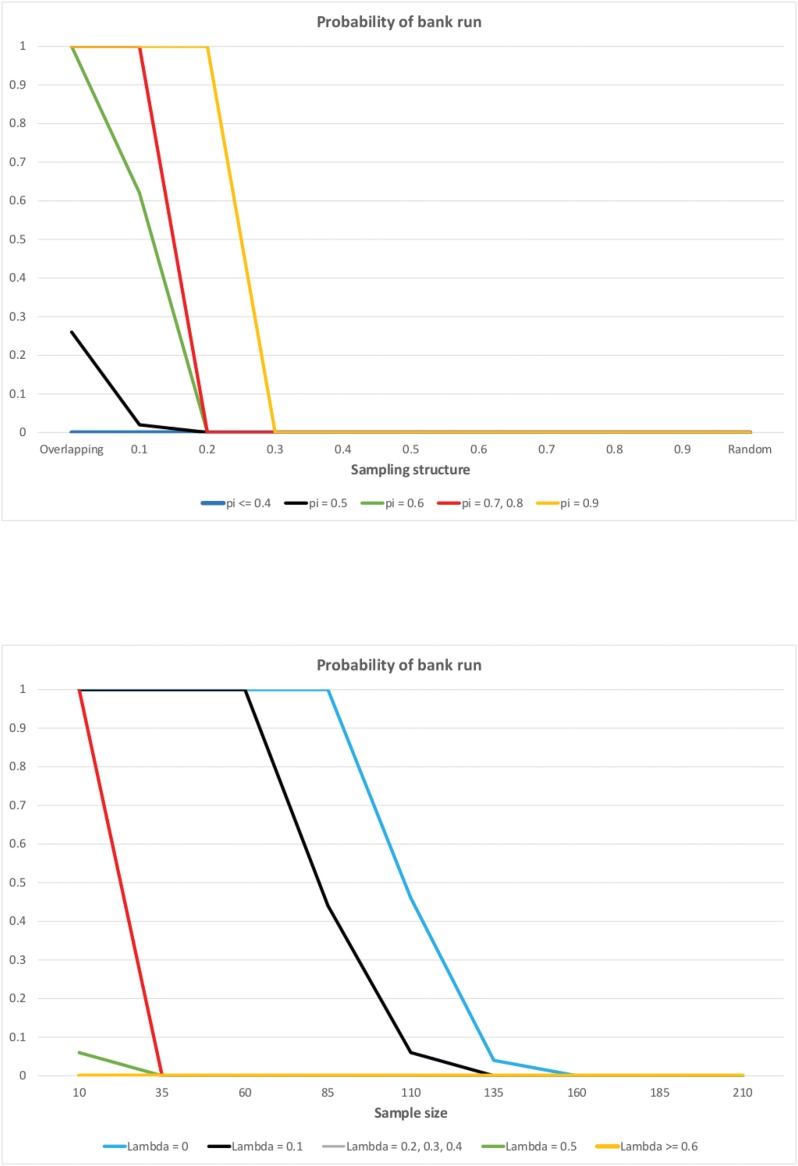
The probability of bank run as the function of the sampling structure (first panel) and the sample size (second panel), as computed from the simulations. Each depositor observes λ*N* randomly drawn previous decisions and (1 − λ)*N* randomly drawn decisions out of his *N* predecessors. The probability of bank run (y-axis) is computed as the percentage of simulation runs where a bank run occurred (out of 100 simulation runs). A bank run occurs in a given simulation run if less than 3% of the last 20000 depositors in the line keep their money in the bank. The population consists of 10^7^ depositors. The parameters are set to *R* = 1.1, *δ* = 1.5 (as in Scenario 1). First panel: The x-axis represents λ going from 0 (overlapping case) to 1 (random case). *N* = 60 and *π* is varied between 0.1 and 0.9. Second panel: The x-axis represents the sample size *N* going from 10 to 210. *π* = 0.7 and the sampling structure λ is varied between 0 (overlapping case) and 1 (random case).

As a robustness check of these findings, we also consider an alternative implementation for the intermediate case. Depositors observe *λN* randomly chosen previous decisions, just as before, and (1 − λ)*N* *randomly drawn decisions out of their* *N* *direct predecessors*, instead of their (1 − λ)*N* direct predecessors as above. The results are shown on Fig 5. It is apparent from the graphs that changing the assumptions on the sampling pattern has no impact on the probability of bank runs and the relationship between this probability and the parameter values λ, *N* and *π*. Fig 5 looks almost exactly the same as Fig 4.

Notice that correlation in the observations and the number of depositors that a bank has may be correlated as well. We argued in the Introduction that clients of a rural bank who live in a close-knit community are more probable to have higher observational correlation than customers of a big bank. If such correlation exists, then based on the previous results we expect more bank runs in small communities with higher observational correlation. However, if the number of depositors is limited, then as discussed at the end of section 3.2, bank runs may not start in spite of large correlation in observations (at least for some parameter values).

## 5 Conclusion

In the canonical [15] model depositors play a simultaneous-move game and decide without observing decisions of other depositors. There are multiple equilibria in this setup, both bank runs and no bank runs are equilibrium outcomes. It is of interest to know which outcome is likelier to occur and which factors affect the emergence of a given outcome.

Empirical research suggests that depositors’ decision is affected by observed previous decisions. The empirical literature also indicates that generally a subset of previous decisions is observed and both type of actions (keeping the money deposited and withdrawal) can be spotted. As the studies analyzing earlier bank run episodes show, these features may lead to bank runs even if the bank does not have fundamental problems. We investigate theoretically and using simulations how the way that the sample is collected influences the probability of bank runs when depositors make overinferences based on the observed sample. The sampling mechanism reflects features of the underlying social structure (e.g. close-knit communities are more likely to exhibit observation structures with high correlation). We find theoretically that when comparing overlapping and random samples, bank runs occur less in the latter case. These findings are corroborated by simulation results that also reveal the importance of the sample size. Hence, by introducing observation of previous decisions in the Diamond-Dybvig framework, by assuming a decision rule often observed in human decision making (the rule of small numbers) and by varying the correlation of observations across depositors we are able to predict to some degree when is a bank run expected to occur. Thus, the inherent multiplicity of the Diamond-Dybvig setup is greatly diminished in our framework.

Policy-makers can affect both the size and the randomness of the sample. Our results suggest that by requiring to give ampler information about depositors’ decision and attempting to make that information less correlated, the probability of bank runs arising from a coordination failure can be efficiently diminished. However, other factors may be relevant also. [41] find experimentally that bank runs are less severe when participants have more information about other subjects’ choices, but only if the economy is in a good state. [20] suggests that during the Great Depression authorities could have bought time to fix problems by limiting public information. Hence, it seems that in good times more information is better, while in bad times the opposite is true. However, in both cases less correlated information may be helpful to prevent bank runs that are not fundamentally justified.

There are still many open questions. As our findings suggest, the underlying social network may determine the correlation across samples. Hence, a fully developed diffusion model in social networks would possibly yield more insights about how information affects depositors’ decisions and the emergence of bank runs.

## Supporting Information

S1 File S1_File.7z contains the program code of all simulations in this paper. The simulations are programmed in Repast 3, an agent-based modeling toolkit available at http://repast.sourceforge.net/repast_3_/. To run the program, one needs a Java Builder, such as Eclipse (available at https://eclipse.org/). After downloading these two programs, a new Java project in Eclipse needs to be created and the two files (Main.java and Agent.java) need to be imported. The downloaded Repast.jar has to be added to the Building path. The main class to run the program is uchicago.src.sim.engine.SimInit. The program generates the output in a txt file (data.txt), it’s location can be specified in line 86 of the program. The output contains the long-run share of depositors who do not withdraw (k*). The parameters can be specified either in the setup method (line 53) or using the Repast GUI that pops up after running the code.
(7Z) Click here for additional data file.
